# Are primary care practitioners in Barbados following hypertension guidelines? - a chart audit

**DOI:** 10.1186/1756-0500-3-316

**Published:** 2010-11-22

**Authors:** O Peter Adams, Anne O Carter

**Affiliations:** 1 Faculty of Medical Sciences, The University of the West Indies, Cave Hill Campus, St. Michael, Barbados; 2 Department of Community Health and Epidemiology, Queen's University, Ontario, Canada

## Abstract

**Background:**

About 55% of the population 40 to 80 years of age in Barbados is hypertensive. The quality of hypertension primary care compared to available practice guidelines is uncertain.

**Findings:**

Charts of hypertensive and diabetic patients were randomly sampled at all public and 20 private sector primary care clinics. Charts of all hypertensive patients ≥ 40 years of age were then selected and processes of care and blood pressure (BP) maintenance < 140/90 documented.

343 charts of hypertensive patients (170 public, and 173 private) were audited. Patients had the following characteristics: mean age 64 years, female gender 63%, mean duration of diagnosis 9.1 years, and diabetes diagnosed 58%. Patients had an average of 4.7 clinic visits per year, 70% were prescribed a thiazide diuretic, 42% a calcium channel blocker, 40% an angiotensin receptor blocker, and 19% a beta blocker. Public patients compared to private patients were more likely to be female (73% vs. 52%, p < 0.01); have a longer duration of diagnosis (11.7 vs. 6.5 years, p < 0.01), and more clinic visits per year (5.0 vs. 4.5, p < 0.01). Over a 2 year period, the proportion of charts with the following recorded at least once was: BP 98%, weight 80%, total cholesterol 71%, urine tested for albumin 67%, serum creatinine 59%, dietary advice 55%, lipid profile 48%, exercise advice 45%, fasting blood glucose for non-diabetics 39%, dietician referral 21%, tobacco advice 17%, retinal examination 16%, body mass index 1%, and waist circumference 0%. Public patients were more likely to have recorded: weight (92% vs. 68%, p = < 0.01); tests for total cholesterol (77% vs. 67%, p = 0.04), albuminuria (77% vs. 58%, p = < 0.01), serum creatinine (75% vs. 43%, p < 0.01), and fasting blood glucose for non-diabetics (49% vs. 30%, p = 0.02); dietician referral (34% vs. 9%, p < 0.01), and tobacco advice (24% vs. 10%, p < 0.01). Most (92%) diastolic BP readings ended in 0 or 5 (72% ended in 0). At the last visit 36% of patients had a BP < 140/90 mmHg.

**Conclusions:**

Improvements are needed in following guidelines for basic interventions such as body mass assessment, accurate BP measurement, use of thiazide diuretics and lifestyle advice. BP control is inadequate.

## Background

Hypertension causes a significant burden of disease in Barbados, with 55% of the black population 40 to 80 years of age estimated to be hypertensive [1]. Most hypertension care in Barbados is done by general practitioners either at no cost to the patient in a public sector polyclinic, or for a fee by a private practitioner. An appropriate range of medication is available to patients at no cost in both sectors.

Previous studies estimated that between 17 and 34% of people being treated for hypertension in Barbados had their blood pressure controlled to < 140/90 [1-3]. A 1994 audit of 3 public clinics, and 5 private general practitioners showed that 18% of persons being treated for hypertension had their blood BP controlled to < 140/90, with this rate being higher in public sector patients [2]. There was no record of lifestyle interventions such as diet and exercise in the majority of charts, and a poor quality of BP measurement was indicated by 92% of readings ending in 0 or 5. In an attempt to produce a higher quality of care the Commonwealth Caribbean Medical Research Council now called the Caribbean Health Research Council (CCMRC and CHRC respectively) developed practice guidelines, Managing Hypertension in Primary Care in the Caribbean, in 1998 [4]. It was distributed to all primary care doctors, nurses, and other health care personnel involved in the care of patients with hypertension in the region, and accompanied (in Barbados) by two seminars for primary care doctors - only a modest intervention support strategy. All published estimates of BP control have been based on data collected prior to the release of these guidelines. A revised version of the CHRC hypertension guidelines was published in 2006, and disseminated in early 2007 [5] again without strong implementation strategies.

This study was carried out in 2005, prior to the release of the revised CHRC hypertension guidelines. The aims of this study were to evaluate the actual status of care of persons with hypertension in primary care in Barbados by means of a chart audit, to determine how closely this care adhered to the 1998 CCMRC guidelines, and to provide baseline data to allow the effectiveness of the 2006 guidelines to be judged in the future. As patient, practitioner and system barriers to guideline implementation may differ between the private and public health sectors a comparison of guideline adherence between sectors is made.

## Methods

### Setting

The population of Barbados was 268,792 at the last census in 2000, of which 95.6% were of African origin [6]. The island is 430 km^2^, and has a good road network. Eight public sector polyclinics strategically located around the island provide free comprehensive primary care, while in 2005 at least 89 private general practitioners were providing service for a fee. All Barbadians are entitled to public sector care. Robust data is not available but it has been estimated that primary care is approximately equally split between the public and private sectors [7].

At public sector polyclinics hypertensive patients are often seen by a nurse who may record the BP, weight and urine test results, before the consultation with the general practitioner. A dietician and podiatrist are available at each clinic on specific days. All polyclinics have a pharmacy. Most private practitioners work in solo or small group practices, and do not employ a nurse. Many patients seen privately do not have health insurance, but drugs for the treatment of hypertension are provided free even to private patients by the Barbados Drug Service. Blood pressure is almost exclusively estimated with mercury sphygmomanometers.

### Chart audit instrument

A chart audit instrument was developed to measure the quality of patient care using indicators recommended in the Managing Hypertension in Primary Care in the Caribbean guidelines [4]. Indicators used included: each visit documentation of weight and blood pressure; annual documentation of urine protein, lipid profile, fasting blood glucose, creatinine and electrolytes; retinal assessment every 2 years; and documented maintenance of BP below 140/90. Since the guidelines recommend that all BP measurements should be recorded to the nearest 2 mm/Hg and at least 2 measurements be taken at each visit, bendrofluazide a thiazide diuretic is the drug of first choice, and treatment recommendations include lifestyle modification (diet, exercise, alcohol and tobacco), indicators to assess adherence to these recommendations were also included. For each patient age, sex, duration of disease, co-morbidity, medications, and health care source (private or public sector) were recorded. No identifying information was collected concerning patients, or health care providers. Data were collected for a two-year period ending on date of the last entry into the chart for hypertension. Processes of care were assessed by noting if they were documented at least once over a 2-year period. For items recommended to be done yearly, the 2-year interval allowed for reasonable delays such as return of lab results and delayed appointments.

A manual explaining the methods of data collection was developed and 3 primary care nurses were trained in random chart sampling, and in data collection.

### Chart sampling

Inclusion criteria were: at least one visit for hypertension in the last year by a patient at least 40 years of age; and hypertension documented in the chart at least 2 years prior to that visit. For the public sector, 100 charts were selected overall, with the number selected at each polyclinic in proportion to the number of patients seen annually by that polyclinic. Private physicians were selected from a previously validated list containing 89 names and asked to participate in a focus group study on diabetes and hypertension. At the end of the focus group session physicians were asked to participate in a chart audit. Only one refused. An additional 9 physicians who could not attend focus groups because they were not available at the time the sessions were held were recruited to make a total of 20 physicians. Since the annual number of patients seen by individual private physicians is unknown, the 20 physicians were asked to contribute 5 charts each. Charts were selected randomly at the various sites. An equal number of charts of patients with diabetes mellitus were also selected in the same way as part of a similar study on diabetes care, and those who had hypertension were also included in this study.

### Data analysis

Data were coded and double entered into SPSS and errors corrected. Proportions of cases meeting the quality indicators were determined. Demographic and disease factors associated with meeting these indicators were determined. With 100 cases from the polyclinics and the same number from the private sector, proportions of cases meeting the quality indicator can be calculated with a 95% confidence interval of < ± 10% in each setting, and < ± 7% for both settings combined. There was > 80% power to detect a 20% difference in prevalence between the two settings.

### Ethical approval

Approval was obtained from the Institutional Review Board of the University of the West Indies, Cave Hill Campus and the Ministry of Health, Barbados.

## Results

A total of 343 charts of hypertensive patients were audited (199 selected because the patient was hypertensive and 144 because the patient was diabetic but also had hypertension), 170 from 8 public polyclinics and 173 from 20 private practitioners. One public clinic contributed 1 less hypertensive chart and 1 less diabetes chart than the required number.

The mean age of patients was 64 years, the mean duration of diagnosis was 9.1 years, 63% were female, and 58% were diabetic. Patients had an average of 4.7 clinic visits per year, 70% were prescribed a thiazide diuretic, 42% a calcium channel blocker, 40% an angiotensin receptor blocker, and 19% a beta blocker. Patients attending public clinics were more likely than private patients to be female; be diagnosed with hypertension for a greater duration; make more frequent clinic visits; have vascular disease and osteoarthritis; and to be prescribed beta blockers. Private physicians were more likely to prescribe angiotensin receptor blockers, and their patients were significantly heavier. Otherwise the patients at the two types of clinics were similar (Table 1).

**Table 1:** Characteristics of hypertensive patients according to whether they attended public or private clinics (n = 343)

Categorical Characteristic	Number with characteristic	Proportion (%)	Proportion (%) attending public clinic N = 170	Proportion (%) attending private clinic N = 173	P value by Chi Square
Gender:% male	128	38	27	48	< .01
% female	213	63	73	52	
Have diabetes*	54	27	27	27	1.00
Have vascular disease	43	13	18	8	< .01
Have heart failure	17	5	6	4	.43
Have osteoarthritis	64	19	27	11	< .01
On thiazide†	240	70	70	70	.99
On indapamide‡	89	26	24	28	.31
On beta blocker	64	19	23	15	.04
On calcium channel blocker	145	42	42	42	.98
On ACE inhibitor§	138	40	39	42	.57
On alpha blocker	14	4	3	5	.29
On centrally acting BP drug	38	11	13	9	.28
On vasodilator	3	1	1	1	.57
On ARBװ	40	12	6	17	< .01
On statin	79	23	24	22	.64
On aspirin	58	17	16	18	.62

**Continuous Characteristic**	**Number with valid data**	**Mean (SD)**	**Mean attending public clinic**	**Mean attending private clinic**	**P value by t-test**

Age (years)	342	64(12)	66	63	.08
Weight (kg)	274	82(21)	77	87	< .01
Clinic visits/yr	340	4.7(1.8)	5.0	4.5	< .01
Years since clinic diagnosis	333	9.1(6.1)	11.7	6.5	< .01

*Includes only the 199 patients included because they were hypertensive, not the 144 who were in the study because they were diabetic but also had hypertension

†Includes the thiazide like diuretics, indapamide and chlorthalidone

‡Almost exclusively prescribed as a brand name product costing > $0.15 per tablet compared to bendrofluazide at $0.01 per tablet.

§Angiotensin Converting Enzyme

װAngiotensin Receptor Blocker

Most patients did not have a recording during a 2-year period of the following items: physical examination (height, body mass index (BMI), waist circumference, and retinal examination); lifestyle (alcohol use, tobacco use and exercise history or advice); laboratory determination of fasting glucose (for non-diabetics) and lipid profile; and referral to a dietician (table 2). Public patients compared to private patients were more likely to have recorded in their charts over a 2-year period the following items: weight, total cholesterol, fasting glucose (for non-diabetics), electrolytes, serum creatinine, urine albumin and history and/or advice on alcohol and tobacco use, and referral to a dietician. Of those on a diuretic, ACE inhibitor or ARB, 59% had an electrolyte and 62% a creatinine done in 2 years. For patients without diabetes 60% had a lipid profile, and 71% a total cholesterol in 2 years.

**Table 2:** Number and proportion of hypertensive patients having process of care recorded at least once in last 2 years according to whether they attended public or private clinic (n = 343 unless otherwise stated)

Recorded process of care	Number meeting require-ment	Proportion (%)	Proportion (%) attending public clinic N = 170	Proportion (%) attending private clinic N = 173	P value by Chi Square
Height recorded*	10	3	2	4	.21
Weight recorded	274	80	92	68	< .01
BMI† recorded	2	1	1	0	.24
WC‡ recorded	0	0	0	0	NA
BP§ recorded	335	98	97	98	.50
Cholesterol done	245	71	77	67	.04
Lipid profile done	166	48	51	46	.31
Fasting glucose doneװ n = 145	57	39	49	30	.02
Creatinine done	202	59	75	43	< .01
Electrolytes done n = 306	176	57	74	42	< .00
Retina assessment recorded	55	16	15	17	.71
Urine albumin recorded	231	67	77	58	< .01
Alcohol recorded	66	19	25	13	< .01
Tobacco recorded	58	17	24	10	< .01
Diet advice recorded	187	55	59	50	.11
Referred to dietician	73	21	34	9	< .01
Exercise recorded	153	45	46	43	.64
All BP's recorded ended in 0	139	40	44	37	.18
All BP's recorded ended in 0 or 5.	261	76	79	73	.24

*Recorded at any time in the chart

†Body Mass Index

‡Waist circumference

§Blood pressure

װDiabetics excluded

Since weight and blood pressure are recommended to be taken at each visit, and visits should occur every quarter, according to the guidelines, a patient should have at least 4 recorded per year or 8 in 2 years (table 3). The remaining variables in table 3 with the exception of retinal assessment are recommended every year or, on average, 2 in 2 years. Retinal assessment is recommended once in every 2 years. Only recording of blood pressure and urine albumin testing on average, met the target. Private patients had, on average, fewer recordings than public patients of weight and urine albumin. Seventy two percent of all blood pressures recorded ended in a 0 (implying rounding to the nearest 10) and 92% ended in either a 5 or a 0 (implying rounding to the nearest 5 or 0) although the guidelines recommended rounding to the nearest 2. For visits at which the BP was recorded, 67% had only a single reading recorded.

**Table 3:** Means of continuous characteristics of hypertensive patients according to whether they attended public or private clinic

Characteristic	Mean (SD) for all patients	Mean for public patients	Mean for private patients	P value by t test	Number with valid data
# hypertension medications	2.1(1.1)	2.1	2.1	.97	343
Total # medications	3.7(1.7)	3.6	3.7	.57	342
# Medication doses/day	4.5(2.6)	4.6	4.4	.55	340
# Weight recorded/yr	2.8(2.0)	3.2	2.2	< .01	342
# Visits with at least 1 BP recorded/yr	4.4 (1.5)	4.6	4.3	.10	342
# BP done by doctor	2.9(2.2)	1.8	4.1	< .01	342
# Diastolic BP/yr end in 0*	3.4((1.6)	3.7	3.2	< .01	341
# Diastolic BP/yr end in 5*	.84(1.2)	0.82	.85	.84	339
# Fasting blood glucoses/yr	.95(1.2)	0.87	1.0	.20	335
# Creatinines/2yr	1.1(1.1)	1.4	0.74	< .01	340
# electrolytes/2yr	1.02(1.2)	1.4	0.68	< .00	306
# Cholesterols/2yr	1.2(1.0)	1.3	1.0	< .01	341
# lipid profiles/2yr	1.0(1.1)	1.2	.90	< .01	336
# Retinal assessments/2yr	.33(.64)	0.40	.27	.16	197
# urine albumin/2yr	2.5(2.9)	3.2	1.9	< .01	187

*Multiple BPs recorded at a single visit, are counted separately.

Table 4 indicates the proportion of patients meeting quality targets. Thirty six percent of patients had their blood pressure controlled at the last visit to < 140/90. Few patients met the target frequency for recording of weight, fasting blood glucose and creatinine, despite 83% having 4 or more visits per year.

**Table 4:** Proportions of hypertensive patients meeting quality targets* at last visit according to whether they attended public or private clinic

Target	Number with valid data	Number meeting target	Proportion (%) meeting target	Proportion (%) attending public clinic who met target	Proportion (%) attending private clinic who met target	P value by Chi Square for difference between public and private
Systolic BP < 140	335	134	40	43	37	.26
Diastolic BP < 90	335	229	68	64	73	.07
Both Systolic BP < 140 and diastolic BP < 90	335	121	36	38	35	.59
BP recorded at 4 or more visits/yr	342	265	78	77	78	.85
4 or more weights recorded/yr	342	134	39	48	31	< .01
2 or more creatinine recorded/2yr	340	110	32	40	25	< .01
2 or more electrolytes recorded/2yr	306	88	29	36	22	< .00
4 or more visits in 1 yr	340	282	83	83	83	.88

*As defined in Managing Hypertension in Primary Care^4^

## Discussion

Barbadian hypertensive patients have adequate access to physicians with an average of 4.7 clinic visits per year, but there are significant deficiencies in the frequency with which several recommended processes of care were performed - history taking, lifestyle advice, physical examination, and laboratory tests - and in the achievement of BP control. Not all factors influencing quality of care could be measured by this study.

### Body mass and lifestyle modification

Height and BMI were recorded in fewer than 4% of charts even though patients were weighed 2.8 times per year. Barbadian patients possibly underestimate their level of overweight and obesity [8] making it imperative that these additional parameters be measured and discussed with them. No chart had a recording of waist circumference, but it should be noted that this was not a CCMRC 1998 guideline requirement.

There was a poor record of the occurrence of lifestyle modification such as weight loss, regular physical activity, and dietary modification which have been shown to reduce BP [9]. Dietician referral was recorded in 21% of charts. Whether the low referral rate was due to physician, patient or system factors could not be determined by this study. Public sector patients were more likely to be referred to a dietician than private patients. Differences in patient characteristics, e.g. public sector patients were more likely to be female, may explain this. In addition private patients unlike public patients have to pay for a dietician who is unlikely to be located in their doctor's office.

### Blood pressure

On average patients had 4.7 different BPs recorded per year, representing a significant investment in time for both health care worker and patient. However almost all BP readings were rounded to 0 or 5 as in the previous audit [2], and usually only one reading was recorded at a clinic visit. This is against guideline recommendations [4]. An inappropriate excess of rounding off to 0 has been reported by multiple investigators [10,11], and could be the most common manifestation of sub-optimal BP measurement [12]. Treatment decisions based on inaccurate readings might result in less than optimal care. It has been estimated that a systematic error in under or over-estimating true BP by 5 mm Hg would cause 21 million Americans to be misclassified as non-hypertensive, and 27 million as hypertensive respectively [13].

Thirty six percent of patients had a blood pressure < 140/90 mm/Hg, compared to 34% of treated hypertensives in a randomly selected population cohort studied from1988 [1]. A 1994 primary care chart audit [2] estimated a control rate of only 18%. However that audit was restricted to 3 polyclinics and 5 private sector general practitioners. A similar chart audit restricted to the same number of clinics and to diabetic patients being treated for hypertension gave a control rate of 17% [3]. In comparison the 1999-2002 National Health and Nutrition Examination Survey estimated that in the USA for patients 40 to 59, and ≥ 60 years of age, 66% and 48% respectively of those on treatment are controlled to a BP of < 140/90 mm Hg [14]. Diastolic pressure was more likely to be controlled than systolic pressure, reflecting the fact that it is often more difficult to control the systolic pressure [9].

### Laboratory tests

Over 40% of patients did not have a serum creatinine and electrolytes done in a 2 year period. Similarly 52% did not have a lipid profile. Cost may be a factor in the private sector as these 2 tests each cost about US $45. In the public sector tests are free, but availability may be a factor for lipid profile testing. The public laboratory sometimes reports only the total cholesterol if there is insufficient reagent to complete a profile. The extent to which this might have been a problem during the study period is not known. Creatinine and electrolytes are usually available in both sectors. The public sector did better than the private sector in testing for proteinuria, possibly reflecting the fact that private physicians unlike the public clinics usually do not employ a nurse.

Twenty seven percent of the patients (mean age 64 years) selected on the basis of hypertension were also diagnosed with diabetes, compared to 18% of a population-based cohort (mean age 59 years) [1]. Only 39% of non-diabetics had a screening fasting blood glucose done in a 2-year period. It is possible that some had a random blood glucose as this can be done more easily, and may be followed by a fasting glucose if equivocal.

### Hypertension treatment

The most frequently used anti-hypertensive agent was the thiazide diuretic. The guidelines [4] recommend starting patients with this class of drugs. ARB's were not available when the guidelines were produced. The JNC 7 guidelines published in 2003 also recommends that treatment should usually be started with a thiazide diuretic [9]. It therefore might be expected that even more patients should have been on this medication, which costs less than US one cent per day unless brand name Indapamide (which costs US 15 cents per tablet) is used [15]. Indapamide was used frequently (37% of patients taking a thiazide were on indapamide) thus reducing the cost benefit of using thiazide diuretics. Switching the 89 patients in this study found to be on indapamide to a generic thiazide would save about US $5000 per year, and extrapolating to the treated hypertensive population would save several hundred thousand dollars.

### Limitations

Some aspects of the sampling method could have introduced bias. Private physicians were not selected on a strictly random basis, and as their patient load is unknown, the quota sampled from each physician could not be weighted. The distribution of care between private and public sectors is also not precisely known. As the sample included charts of patients who were diabetic but also had hypertension, a higher proportion of charts of diabetic patients were audited than if selection was on the basis of hypertension only (58% vs. 27%). In addition chart audits are affected by the completeness of the recording as well as the accuracy with which recorded measurements are done. Lifestyle measures might be affected more than other processes by not being recorded. Inaccurate BP measurement would result in an inaccurate estimate of BP control.

## Conclusions

Despite the creation and distribution of guidelines 6 years before the study period began, a significant gap between the guidelines and actual practice exists. This supports the evidence that guidelines must be accompanied by intensive, multi-pronged implementation efforts if they are to achieve improved quality of care [16,17]. Improving compliance with guidelines for many of the recommended interventions may have varying financial implications, and requires different strategies. Health care workers have to be provided with the resources needed to facilitate compliance, and care has to be affordable to private patients. Some simple office procedures such as measuring height, weight, urine albumin, taking adequate histories and giving advice cost only the increase in staff time required to do them. Repetitive retraining in auscultation, or the use of validated automated oscillometric BP monitors can reduce the observer error demonstrated in BP measurement by this study. Cost effective prescribing might partially balance the expense of implementing these changes.

## Competing interests

The authors declare that they have no competing interests.

## Authors' contributions

OPA, AOC participated in the conception and design of the study; the acquisition, and interpretation of data; and drafting and revising the manuscript critically. AOC analysed the data. Both authors have read and approved the final manuscript.
